# Causes and Consequences of miR-150-5p Dysregulation in Myasthenia Gravis

**DOI:** 10.3389/fimmu.2019.00539

**Published:** 2019-03-29

**Authors:** Mélanie A. Cron, Solène Maillard, Frédérique Truffault, Ambra Vittoria Gualeni, Annunziata Gloghini, Elie Fadel, Julien Guihaire, Anthony Behin, Sonia Berrih-Aknin, Rozen Le Panse

**Affiliations:** ^1^Center of Research in Myology, Sorbonne University, INSERM, Association Institute of Myology - UMRS 974, Paris, France; ^2^Department of Pathology and Laboratory Medicine, Istituto Nazionale dei Tumori, Milan, Italy; ^3^Marie Lannelongue Hospital, Paris-Sud University, Le Plessis-Robinson, France; ^4^Neuromuscular Disease Center, AIM, Pitié-Salpêtrière Hospital, AP-HP, Paris, France

**Keywords:** autoimmunity, germinal center, antimir-150, MYB, microRNA

## Abstract

Autoimmune Myasthenia gravis (MG) is a chronic neuromuscular disease mainly due to antibodies against the acetylcholine receptor (AChR) at the neuromuscular junction that induce invalidating muscle weaknesses. In early-onset MG, the thymus is the effector organ and is often characterized by B-cell infiltrations leading to ectopic germinal center (GC) development. The microRNA miR-150-5p has been previously characterized as a biomarker in MG due to its increase in the serum of patients and its decrease after thymectomy, correlated with an improvement of symptoms. Here, we investigated the causes and consequences of the miR-150 increase in the serum of early-onset MG patients. We observed that miR-150 expression was upregulated in MG thymuses in correlation with the presence of thymic B cells and showed by *in situ* hybridization experiments, that miR-150 was mainly expressed by cells of the mantle zone of GCs. However, we did not observe any correlation between the degree of thymic hyperplasia and the serum levels in MG patients. In parallel, we also investigated the expression of miR-150 in peripheral blood mononuclear cells (PBMCs) from MG patients. We observed that miR-150 was down-regulated, especially in CD4^+^ T cells compared to controls. These results suggest that the increased serum levels of miR-150 could result from a release from activated peripheral CD4^+^ T cells. Next, we demonstrated that the *in vitro* treatment of PBMCs with miR-150 or antimiR-150 oligonucleotides, respectively, decreased or increased the expression of one of its major target gene: the proto-oncogene *MYB*, a well-known actor of hematopoiesis. These results revealed that increased serum levels of miR-150 in MG patients could have a functional effect on PBMCs. We also showed that antimiR-150 caused increased cellular death of CD4^+^ and CD8^+^ T cells, along with the overexpression of pro-apoptotic genes targeted by miR-150 suggesting that miR-150 controlled the survival of these cells. Altogether, these results showed that miR-150 could play a role in MG both at the thymic level and in periphery by modulating the expression of target genes and peripheral cell survival.

## Introduction

Myasthenia Gravis (MG) is an autoimmune disease due to antibodies against several components of the neuromuscular junction. Patients suffer from more or less invalidating muscle weaknesses leading to a generalized fatigability (1). The majority of patients (85%) display antibodies against the acetylcholine receptor (AChR). Moreover, in the early-onset form of the disease, functional and morphological thymic abnormalities are frequently observed. They are mainly characterized by active neoangiogenic processes (2, 3) leading to B-cell infiltrations associated with the ectopic development of germinal centers (GCs) (4): thymic hyperplasia (5). There is no global correlation between the AChR antibody titer and the severity of the disease, although such a correlation has been described at the individual level (6). A clear correlation exists between the anti-AChR antibodies serum level and the degree of thymic hyperplasia (7). Moreover, the number of GCs is reduced in patients undergoing corticosteroid treatment (8). All these observations support the role of the thymus in the pathogenesis of MG, and thymectomy is often advised for AChR-MG patients to improve symptoms (9).

In collaboration with Dr. Punga's group, we investigated microRNAs (miRNAs) as potential circulating biomarkers in early-onset AChR-MG patients. We observed that miR-150-5p (miR-150) is upregulated in the serum of AChR^+^ patients and its expression is lowered after thymectomy, accompanied by an improvement of symptoms (10). Another study on late-onset MG patients showed a negative correlation between the expression of circulating miR-150 and the improvement of patients' clinical status (11).

First described in 2007 by Zhou et al. in hematopoietic stem cells (12), miR-150 is an immuno-miR regulating immune functions, such as proliferation, apoptosis and differentiation of NK, T and B cells (13–17), as well as central tolerance. miR-150 is also a marker of lymphocyte activation (18) and is highly sensitive to stress signals such as lipopolysaccharides or glucocorticoids (19, 20). As in early-onset MG patients, miR-150 serum level is increased in various immune pathologies as in multiple sclerosis (21), in irritable bowel syndrome (22), during HIV infection (23) but also in some cancers (24, 25). Of interest, miR-150 is also overexpressed in inflammatory organs, as for example in salivary glands in Sjögren Syndrome (26) and in kidneys in lupus nephritis (27). Overall, miR-150 is thought to be a sensor of general lymphocyte activation induced by inflammation (28).

In this study, we investigated the expression levels of miR-150 in the thymus and in peripheral blood cells of AChR-positive MG patients in order to better understand the increase observed in the serum. We observed that miR-150 was increased in the MG thymus in correlation with the presence of B cells, and decreased in peripheral blood cells, especially in CD4^+^ T cells. In addition, we demonstrated that serum level of miR-150 could have a functional role on peripheral blood cells by controlling the expression of MYB or pro-apoptotic genes, such as P53 and AIFM2.

## Methods

### Thymic and Blood Samples

**Figure 1:**
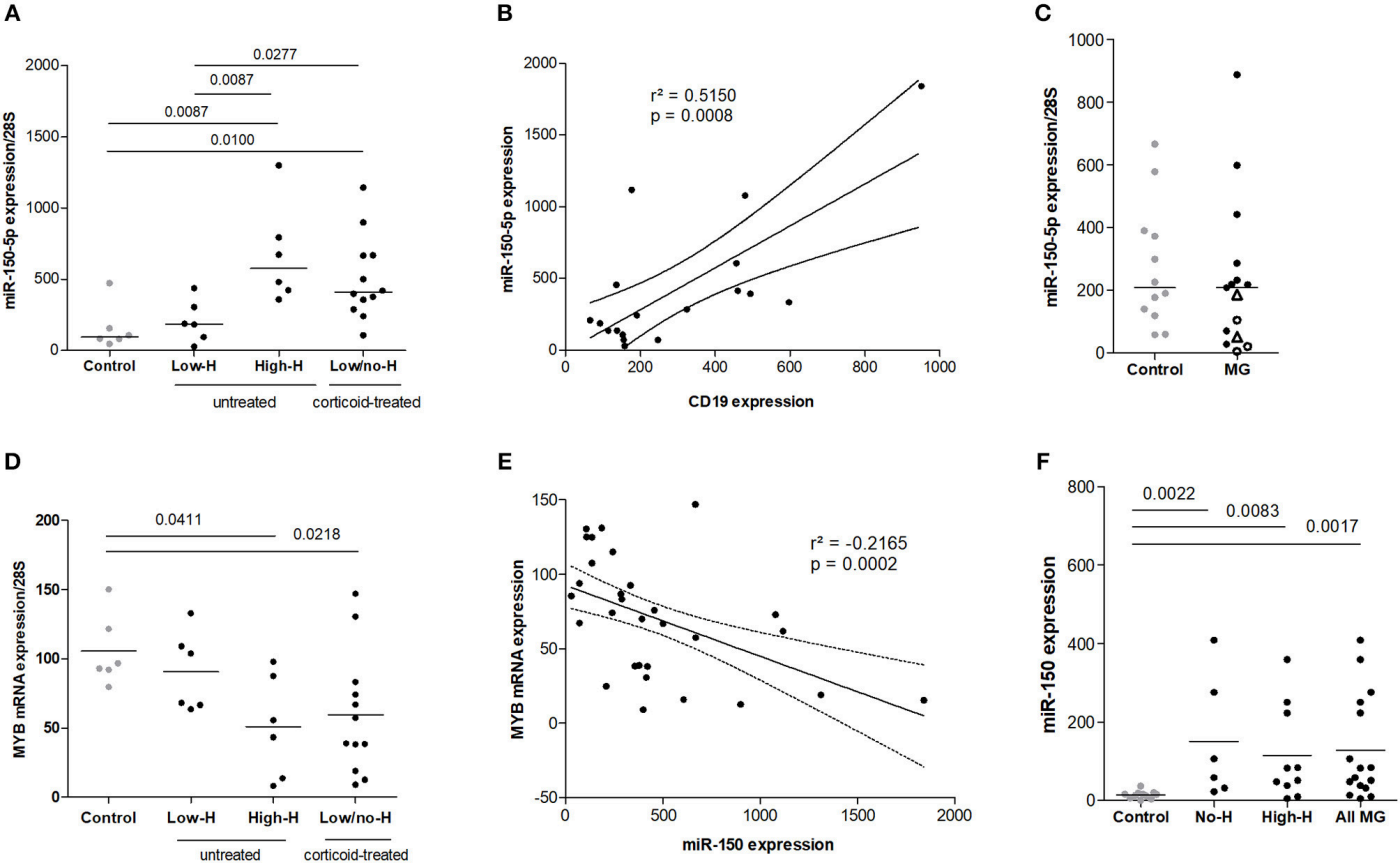
Increased expression of miR-150–5p associated with thymic hyperplasia in MG. **(A)** miR-150 expression in the thymus of non-MG controls (*n* = 6), MG patients with a low degree of thymic hyperplasia (Low-H, *n* = 6) or with a high degree of thymic hyperplasia (High-H, *n* = 6) and in corticoid-treated MG patients displaying low or no hyperplasia (Low/no-H; *n* = 12). miR-150 expression was normalized on 28 S expression. **(B)** Correlation analysis of miR-150 and *CD19* mRNA expression in the thymus of controls (*n* = 6) and untreated MG patients (*n* = 12) **(C)** miR-150 expression in medullary thymic epithelial cells from non-MG adults (*n* = 12) and untreated MG patients (*n* = 15). Thymic epithelial cells from High-H MG patients are represented with empty black dots and cells from patients undergoing corticoid therapy at the time of thymectomy are represented as empty black triangles. **(D)** *MYB* mRNA expression in controls (*n* = 6), untreated MG patients (Low-H, *n* = 6; High-H, *n* = 6) and corticoid-treated MG patients (Low/no-H, *n* = 12). mRNA expression was normalized on 28S expression. **(E)** Negative correlation analysis of miR-150 and *MYB* mRNA expression in the thymus of controls (*n* = 6), untreated (*n* = 12) and cortico-treated MG patients (*n* = 12). **(F)** miR-150 expression in the serum of controls (*n* = 11), MG patients without thymic hyperplasia (No-H; *n* = 6) and MG patients with a high degree of thymic hyperplasia (High-H, *n* = 10). No-H and High-H patients were pooled for the “All MG” category. *P*-values were assessed by the Mann-Whitney test and only *p*-values < 0.05 are indicated on the graphs. Pearson's correlation was assessed and determination coefficient and *p*-values are indicated on the graphs.

Thymic biopsies were obtained from the Marie Lannelongue Surgical Center (Le Plessis-Robinson, France), where early-onset AChR-positive MG patients (15–44 years old, *n* = 40 with details given in Table 1A) underwent thymectomy and age/sex-matched non-MG controls (15–36 years old, *n* = 19) cardiovascular surgery. MG thymuses were classified according to the degree of follicular hyperplasia assessed by Marie Lannelongue Surgical Center pathologists (high degree of hyperplasia with 3 or more GCs per section vs. low degree of hyperplasia with 2 or less GCs per section). This classification was confirmed by the analyses of *CD19* mRNA expression in the thymic biopsies used in Figure 1. Indeed, the expression of *CD19* was significantly higher in the group of untreated-MG patients with a high degree of hyperplasia (mean ± SEM = 388.8 ± 63.9, *n* = 6) compared to the group of untreated MG-patients with a low degree of hyperplasia (mean ± SEM = 188.5 ± 38.3, *n* = 6) or treated MG patients with no or a low degree of hyperplasia (mean ± SEM = 253.7 ± 55.0, *n* = 12). Thymic epithelial cells were extracted from control and MG thymic biopsies from patients and were used for real-time PCR (RT-PCR) as previously described (29).

**Table 1A:** Characteristics of patients whose thymus and TECs were used for experiments.

**Patient ID**	**Degree of thymic hyperplasia (1)**	**Interval onset - thymectomy (months)**	**MGFA score at thymectomy (2)**	**Corticoid treatment (3)**	**Cholinesterase inhibitors**	**Anti-AChR titer (nmol/L)**	**PCR on thymic extracts**	**PCR on TECs**	***In situ* hybridization**
MG1	Low	13	III b	No	Yes	3.45	X		
MG2	Low	7	II a	No	Yes	2118.7	X	X	
MG3	Low	7	I a	No	NS	83.7	X		
MG4	Low	24	IV a	No	Yes	11.1	X		
MG5	Low	6	II a	No	Yes	>100	X		
MG6	Low	6	NS	No	Yes	17.3	X		
MG7	High	2	IV a	No	NS	>100	X	X	
MG8	High	14	I	No	NS	3180.2	X		
MG9	High	3	II a	No	Yes	3.21	X		
MG10	High	4	II a	No	Yes	60.38	X		
MG11	High	36	III a	No	Yes	9.7	X		
MG12	High	2	III a	No	Yes	264	X		
MG13	No hyperplasia	6	IV b	Yes	Yes	30.02	X		
MG14	No hyperplasia	18	III a	Yes	Yes	0.5	X		
MG15	No hyperplasia	30	II b	Yes	Yes	4.33	X		
MG16	No hyperplasia	6	III a	Yes	Yes	3.46	X		
MG17	Low	18	II b	Yes	Yes	NS	X		
MG18	No hyperplasia	18	V	Yes	Yes	6.9	X		
MG19	Low	24	NS	Yes	Yes	4.4	X		
MG20	NS	9	III a	Yes	Yes	9.6	X		
MG21	No hyperplasia	60	NS	Yes	Yes	21.4	X		
MG22	Low	10	II a	Yes	Yes	26.1	X		
MG23	No hyperplasia	12	NS	Yes	Yes	6.6	X		
MG24	No hyperplasia	30	II a	Yes	Yes	NS	X		
MG25	Low	3	III a	No	Yes	14		X	
MG26	High	18	IV a	No	Yes	>100		X	
MG27	Low	34	IV b	Yes	Yes	206.57		X	
MG28	Low	3	II a	No	Yes	>100		X	
MG29	Low	48	III b	No	Yes	0.38		X	
MG30	High	9	NS	No	Yes	NS		X	
MG31	Low	18	IV a	No	Yes	670		X	
MG32	Low	33	NS	Yes	Yes	>100		X	
MG33	Low	26	II a	No	Yes	87.6		X	
MG34	Low	84	II b	No	Yes	35.3		X	
MG35	NS	24	NS	No	Yes	NS		X	
MG36	Low	NS	NS	No	Yes	NS		X	
MG37	Low	6	II a	No	Yes	1.1		X	
MG38	High	4	II a	No	Yes	60.38			X
MG39	High	2	IV a	NS	NS	>100			X
MG40	High	3	II a	No	Yes	3.21			X

*Patients are females (37), aged from 15 to 44, and males (3) aged from 20 to 35. (1) Degree of thymic hyperplasia: low hyperplasia (with 2 or fewer GCs per section) or high hyperplasia (with 3 or more GCs per section). (2) Clinical classification according to the Myasthenia Gravis Foundation of America (MGFA). (3) Patients with corticoid treatment were only used for RT-PCR in Figure 1A. NS, not specified*.

Blood was collected from MG patients in order to isolate peripheral blood mononuclear cells and serum at the time of the thymectomy (PBMCs: 19–44 years old, *n* = 27, Table 1B/serum: 18–46 years old, *n* = 16, Table 1C) and from control donors by the French Blood Establishment (EFS) (PBMCs: 18–49 years old, *n* = 14 / serum: and 23–59 years old, *n* = 11).

**Table 1B:** Characteristics of patients whose PBMCs were used for experiments.

**Patient ID**	**Degree of thymic hyperplasia (1)**	**MGFA score at thymectomy (2)**	**Corticoid treatment**	**Cholinesterase inhibitors**	**Anti-AChR titer (nmol/L)**	**PCR on PBMCs**	**PCR on CD4^**+**^ T cells**	**PCR on CD19^**+**^ B cells**
MG41	NS	NS	No	No	>100	X		
MG42	High	NS	No	Yes	8	X		
MG43	Low	NS	No	Yes	117.9	X		
MG44	High	III b	No	Yes	65.9	X		
MG45	Low	II a	No	Yes	>100	X	X	X
MG46	High	NS	No	Yes	10.9	X	X	X
MG47	Low	NS	No	Yes	12.4	X	X*	X
MG48	Low	NS	No	Yes	7	X	X*	X
MG49	Low	II b	No	Yes	48		X	X
MG50	Low	II a	No	Yes	1.6		X*	X
MG51	Low	II b	No	Yes	1.67		X*	X
MG52	Low	II b	No	Yes	1.82		X*	X
MG53	High	II b	No	Yes	>100		X	
MG54	High	NS	No	Yes	23.3	X		X
MG55	Low	II b	No	Yes	2.3			X
MG56	High	II b	No	Yes	5.8			X
MG57*	Low	II b	No	Yes	1.04	X		
MG58*	NS	III b	No	Yes	NS	X		
MG59*	NS	NS	No	No	2.48	X		
MG60*	NS	NS	No	No	>100	X		
MG61*	Low	NS	No	Yes	1.82	X		
MG62*	High	NS	No	Yes	29	X		
MG63*	NS	NS	No	No	>100	X		
MG64*	Low	NS	No	Yes	1.1	X		
MG65*	Low	NS	No	No	4.9		X	
MG66*	Low	NS	No	Yes	2.3		X	
MG67*	High	NS	No	Yes	5.8		X	

Patients are females (25), aged from 19 to 44, and males (2) aged of 24 and 33. *: patients used for MYB expression analysis on Figures 3D,E. (1) Degree of thymic hyperplasia: low hyperplasia (with 2 or fewer GCs per section) or high hyperplasia (with 3 or more GCs per section). (2) Clinical classification according to the Myasthenia Gravis Foundation of America (MGFA).

**Table 1C:** Characteristics of patients whose serum was used for experiments.

**Patient ID**	**Degree of thymic hyperplasia (1)**	**Anti-AChR titer (nmol/L)**
MG68	High	80
MG69	High	7.6
MG70	High	21.8
MG71	High	10.9
MG72	High	50.9
MG73	High	NS
MG74	High	16.3
MG75	High	14.5
MG76	No hyperplasia	0
MG77	No hyperplasia	0
MG78	No hyperplasia	2.27
MG79	No hyperplasia	1.66
MG80	No hyperplasia	3.7
MG81	No hyperplasia	0
MG82	High	8.7
MG83	High	36

*Patients are females (13), aged from 23 to 46, and males (3) aged of 18 to 37. All patients were under anticholinesterasic therapy and were corticoid-free. (1) Degree of thymic hyperplasia: low hyperplasia (with 2 or fewer GCs per section) or high hyperplasia (with 3 or more GCs per section)*.

MG patients did not display any thymoma or any infectious diseases. All patient information are displayed on Tables 1A-C and studies on blood and thymic samples were approved by local ethics committees (RCB 2006-A00164-47 and RCB 2010-A00250-39).

### *In situ* Hybridization

*In situ* hybridization was performed on formalin-fixed paraffin-embedded (FFPE) thymic tissues (3 adult controls and 3 MG donors (Table 1A)) using a semi-automated method previously described (30). Briefly, 5 μm-thick FFPE thymic tissues on polarized glass slides (Menzel-Gläzer SuperFrost Plus, Thermo Scientific, Villebon-sur-Yvette, France) were placed in xylene and hydrated using ethanol-descending concentration baths. Tissue permeabilization was done using protease-K before dehydration of slides by placing them in ethanol-increasing concentration baths. Previously diluted to 100 nM and denatured double-DIG-LNA probes (Exiqon, Vedbaek, Denmark) for miR-150-5p (#38053-15) and for the scramble miRNA (#99004-15) were placed directly on slides and incubated in a hybridizer (Dako-Agilent, Santa Clara, USA) for 2 h at 53°C for miR-150-5p probe and 57°C for scramble probe. After several stringent washes, the automated protocol was applied. This protocol includes an incubation with peroxidase inhibitor and a blocking step with casein followed by an incubation with mouse anti-DIG primary antibody. Signal detection was performed by OptiviewDAB Detection Kit (#760-700, Ventana Medical Systems, Tucson, USA) and OptiView Amplification Kit (#760-099). Finally, slides were dehydrated and mounted to visualize hybridization with a bright-field microscope.

### PBMCs and Cell Sorting

PBMCs from MG patients and healthy controls were isolated from fresh whole blood, collected in EDTA tubes, using the Ficoll technique (31). Cells were stored in fetal calf serum containing 20% DMSO and kept in liquid nitrogen until use. If possible CD19^+^ B and CD4^+^ T cells were successively sorted with EasySep kits (#18054, #18052, Stem Cell, Vancouver, Canada). Cells were next used for RNA extraction and PCR for miR-150 and 28S. Details on MG patients used for PBMCs, CD19^+^ B, and/or CD4^+^ T cells are given in Table 1B. Only 6 controls and 3 MG samples were used in common for miR-150 and MYB analyses in PBMCs.

For PBMC cultures, cells from healthy donors were seeded in X-vivo medium (Lonza, Levallois-Perret, France) in 96-well plates (1.10^6^ cells per well). Next cells were treated with a non-toxic dose of 10 μM of miR-150, antimiR-150 or a scramble miRNA from *Caenorhabditis elegans* (cel-miR-293b) (Eurogentec, Seraing, Belgium). The natural uptake of naked oligonucleotides by the cells is named gymnosis and occurs without creating pores by transfection or other chemical reagents and physical techniques (32). antimiR-150 is an unmodified miRNA specifically targeting miR-150 in order to inhibit it. miR-150, antimiR-150 and scramble miRNA sequences are available in Table 2A. After 72 h, non-adherent peripheral blood lymphocytes (PBLs) were collected and used for quantitative PCR (qPCR), Western blot or flow cytometry analyses.

**Table 2A:** miRNA sequences used for culture treatments and qPCR.

**Name (miRBase ID)**	**Sequence**
hsa-miR-150-5p (MIMAT0000451)	5′-UCUCCCAACCCUUGUACCAGUG-3′
cel-miR-239b-5p (MIMAT0000295)	5′-UUGUACUACACAAAAGUACUG-3′
antimiR-150 (none)	5′-CACUGGUACAAGGGUUGGGAGA-3′

### RNA Extraction

Total RNA was extracted from thymic biopsies or cells with the mirVana miRNA Isolation Kit in particular for RT-PCR on miRNAs or by TRIzol for mRNA analyses (Life Technologies, Villebon-sur-Yvette, France). Biopsies were first lysed in the Lysis/Binding buffer provided in the mirVana kit with the FastPrep FP120 instrument (Qbiogen, Illkirch, France) whereas cells were directly lysed in TRIzol after culture. RNA was isolated from serum with the miRCURY RNA Isolation Kit Biofluids (Exiqon) according to the manufacturer's instructions. RNA quality was assessed on a Bioanalyzer 2100 (Agilent Technologies, Les Ulis, France).

### RT-PCR for miRNAs

miRNAs were retro-transcribed from total RNA using the Universal cDNA Synthesis Kit II (Exiqon), according to the manufacturer's instructions. cDNAs were next diluted to 1:20 in nuclease-free water prior to qPCR reactions that were carried out using the ExiLENT SYBR Green master mix (Exiqon) on a LightCycler 480 (Roche, Meylan, France). PCR settings were as followed: 1 cycle of polymerase activation and denaturation at 95°C for 10 min, 45 cycles of amplification at 95°C for 10 s and 60°C for 1 min, with a ramp-rate of cooling of 1.6°C/s.

To normalize RT-PCR data, we tested U6 and miR-191, two classical references but their expression was either not good or variable among samples. miRNA expression was then normalized to 28S rRNA expression that was very stable in all samples. miRNA sequences used for RT-PCR are listed on Table 2A.

### RT-PCR for mRNAs

mRNAs were retro-transcribed from 1 μg of total RNA using the Reverse Transcriptase AMV kit (Roche) and qPCR experiments were carried out using LightCycler 480 SYBR Green Master Mix (Roche) according to the manufacturer's instructions. qPCR cycle conditions were: 1 cycle of polymerase activation and denaturation at 95°C for 10 min, 45 cycles of amplification at 95°C for 10 s, 60–64°C for 1 min and 72°C for 14 s. Primer sequences are listed on Table 2B.

**Table 2B:** Primer sequences used for qPCR.

**Name**	**Sequence**	**Orientation**	**Amplicon size (pb)**
28S	5′-GTAGGGACAGTGGGAATCT-3′	Forward	108
	5′-CGGGTAAACGGCGGGAGTAA-3′	Reverse	
AIFM2	5′-CTGAACGTCCCCTTCATGCT-3′	Forward	158
	5′-ATCCCCACTACTAGCCCCTG-3′	Reverse	
GAPDH	5′-CGACCACTTTGTCAAGCTCA-3′	Forward	228
	5′-AGGGGTCTACATGGCAACTG-3′	Reverse	
MYB	5′-TTCCACAGGATGCAGGTTCC-3′	Forward	208
	5′-TGGGAAGGGGACAGTCTGAA-3′	Reverse	
P53	5′-GGCCCACTTCACCGTACTAA-3′	Forward	156
	5′-GTGGTTTCAAGGCCAGATGT-3′	Reverse	
PDCD4	5′-TGTGGAGGAGGTGGATGTGA-3′	Forward	249
	5′-TGACTAGCCTTCCCCTCCAA-3′	Reverse	

### Flow Cytometry

PBMCs were surface-stained with anti-human monoclonal antibodies for 45 min at 4°C: anti-CD4-APC (clone RPA-T4; #17-0049-42, eBioscience, Paris, France), anti-CD8-PeCy7 (clone RPA-T8; #557750, BD Biosciences, Le-Pont-de-Claix, France) and anti-CD19-ef450 (clone HIB19; #48-0199-42, eBioscience) antibodies. Cells were fixed and permeabilized with the Intracellular Fixation and Permeabilization buffer set (#00-5523-00, eBioscience) according to the manufacturer's instructions. Intracellular MYB was stained with an anti-MYB polyclonal antibody (#AF6209, R&D Systems). Live/dead staining (Fixable Near-IR Dead Cell Stain Kit, #L10119, Life Technologies) was done to gate on viable cells and to analyze cell death. A Ki-67-PeCy7 antibody (clone SolA15; #25-5698-82, eBioscience) was used to assess cell proliferation. Flow cytometry was performed on a FACS Canto II (BD Biosciences) and data were analyzed using FlowJo software.

### Western Blot

Whole-cell proteins from PBLs were extracted in RIPA buffer (20 mM Tris HCl pH 8, 137 mM NaCl, 10% Glycerol, 1% NP40, 2 mM EDTA) and cellular debris were eliminated after centrifugation at 12,000 g for 25 min at 4°C. Protein concentration was assessed using the Pierce BCA Protein Assay Kit (Thermo Fisher Scientific) according to the manufacturer's instructions. Fifteen microgram of proteins were diluted in Laemmli buffer, separated on polyacrylamide gels (NuPAGE 4–12% Bis-Tris Gel, Life Technologies) and transferred to a PVDF membrane (Immobilon-P, Merck-Millipore, Darmstadt, Germany). Transfer efficiency was evaluated with Ponceau staining. Next, the membrane was saturated using 5% non-fat milk diluted in TBS 0.05% Tween (TBS-T) for 1 h, incubated with mouse anti-human MYB monoclonal antibody (2 μg/mL, #05-175, Merck-Millipore) overnight at 4°C. After several washes in TBS-T, the membrane was incubated with HRP sheep anti-mouse secondary antibody (#NA931, GE Healthcare, United Kingdom) for 1 h at room temperature. Finally, the membrane was washed in TBS-T and revealed with electrochemiluminescence (ECL) on autoradiography films (Amersham, GE Healthcare). MYB intensity was normalized on β-actin (#612656, BD Transduction Laboratories) expression. Band intensity quantification was done using ImageJ software.

### Statistical Analysis

In correlation analyses, a Pearson's correlation test was applied and significance was assessed. In dot plot graphs, we used the non-parametric Mann Whitney test or the Wilcoxon paired test to do 2-by-2 comparisons, as indicated in figure legends.

## Results

### Increased miR-150 Expression in Highly Hyperplastic Thymus

miR-150 expression levels were assessed in the thymus of early-onset MG patients displaying different degrees of follicular hyperplasia characterized by the number of GCs. miR-150 was particularly increased in untreated MG patients with a high degree of thymic hyperplasia compared to controls and to patients with a low degree of hyperplasia. In a miRnome study analyzing dysregulated miRNAs in the MG thymus, miR-150 was not characterized as up-regulated because patients with a high or low degree of thymic hyperplasia were analyzed altogether (33). However, when taking into account the degree of hyperplasia, miR-150 was also found up-regulated in hyperplastic MG thymuses (data not shown).

In corticoid-treated patients, with a low or no hyperplasia, the levels of miR-150 were not reduced compared to MG patients with a high degree of hyperplasia (Figure 1A). Even if corticoids are well known to decrease the number of GCs (8), corticoids have also the ability to induce miR-150 expression as described by Palagani et al. (20). We did not observe any correlation between the thymic levels of miR-150 and the delay between MG symptom onset and the thymectomy, or the severity of the disease (data not shown).

As we detected a significant overexpression of miR-150 in patients with a high degree of thymic hyperplasia, we analyzed the expression of *CD19* mRNA, a B-cell marker, in correlation with miR-150 expression for controls and untreated-MG patients. We demonstrated a significant positive correlation between *CD19* mRNA and miR-150 expression (Figure 1B). As they represent one of the major cell population in the thymus, we also assessed miR-150 expression in thymic epithelial cells but did not see any differences between controls and MG patients (Figure 1C).

In parallel, we analyzed in MG patients the expression levels of *MYB* mRNA, one of miR-150 best-known and major target. In MG patients, *MYB* was significantly down-regulated in the thymus of untreated MG patients with a high degree of hyperplasia and in cortico-treated MG patients (Figure 1D). More, we showed that *MYB* expression was negatively and significantly correlated with miR-150 expression in the thymus of control and all MG patients (Figure 1E).

Altogether, these results suggest that miR-150 overexpression in untreated MG thymuses is due to B cells and to the presence of ectopic GCs, and that increased expression of miR-150 could modulate intrathymic expression of mRNA targets such as *MYB*.

As previously described, miR-150 expression is increased in the serum of MG patients compared to controls (10). We confirmed that miR-150 was overexpressed in the serum of MG patients and showed that this overexpression was independent of the degree of hyperplasia, as we did not observe any difference between patients without thymic hyperplasia (No-H) and patients with a high degree of hyperplasia (High-H) (Figure 1F).

### Strong miR-150 Expression in B Cells of the Mantle Zone of Germinal Centers

**Figure 2:**
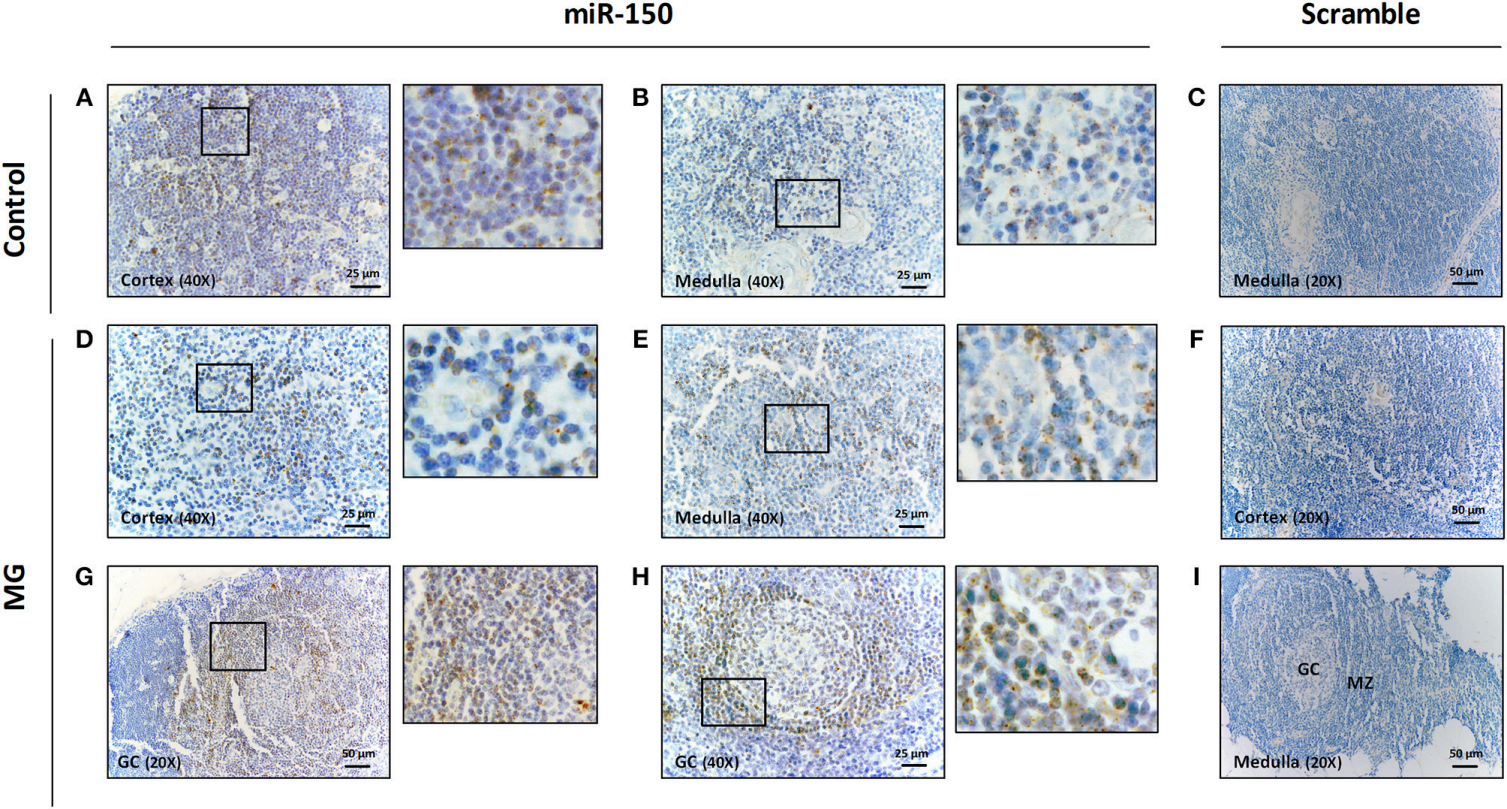
miR-150 was highly expressed in B cells of the mantle zone of GCs in MG thymuses. Representative pictures of *in situ* hybridization of miR-150 in the cortex **(A)** and the medulla **(B)** of the thymus of a non-MG control and in the cortex **(D)**, the medulla **(E)** and two different GCs **(G,H)** of the thymus of MG patients. miR-150 was located in B cells of the mantle zone of ectopic follicle with expanded GC **(G,H)**. Scramble labeling was performed in the medulla **(C)** of the thymus of a non-MG control and in the cortex **(F)** and in the medulla/GC area **(I)** of the thymus of MG patients. 1-cm bar length represents 50 μm for 20X magnification and 25 μm for 40X magnification. All *in situ* hybridization experiments were done in formalin fixed paraffin embedded thymic tissue sections, as fully detailed in the method section.

In order to visualize more precisely the localization of miR-150 in the thymus, we performed *in situ* hybridization in control (Figures 2A,B) and MG (Figures 2D,E and 2G,H) thymuses. A miR-150 labeling was observed associated with thymocytes in both the cortical and medullary regions, and miR-150 distribution seemed similar in adult control and MG patients (Figures 2A,B and 2D,E). No unspecific labeling was observed using a scramble miRNA (Figures 2C,F,I).

However, in the thymus of MG patients, we observed a strong labeling for miR-150 preferentially located in the mantle zone around GCs (Figures 2G,H). These data demonstrated that the higher expression of miR-150 expression as observed by PCR in the hyperplastic thymus of untreated MG patients was linked to B cells and ectopic GCs.

### Decreased Expression of miR-150-5p in peripheral CD4^+^ T Cells

**Figure 3:**
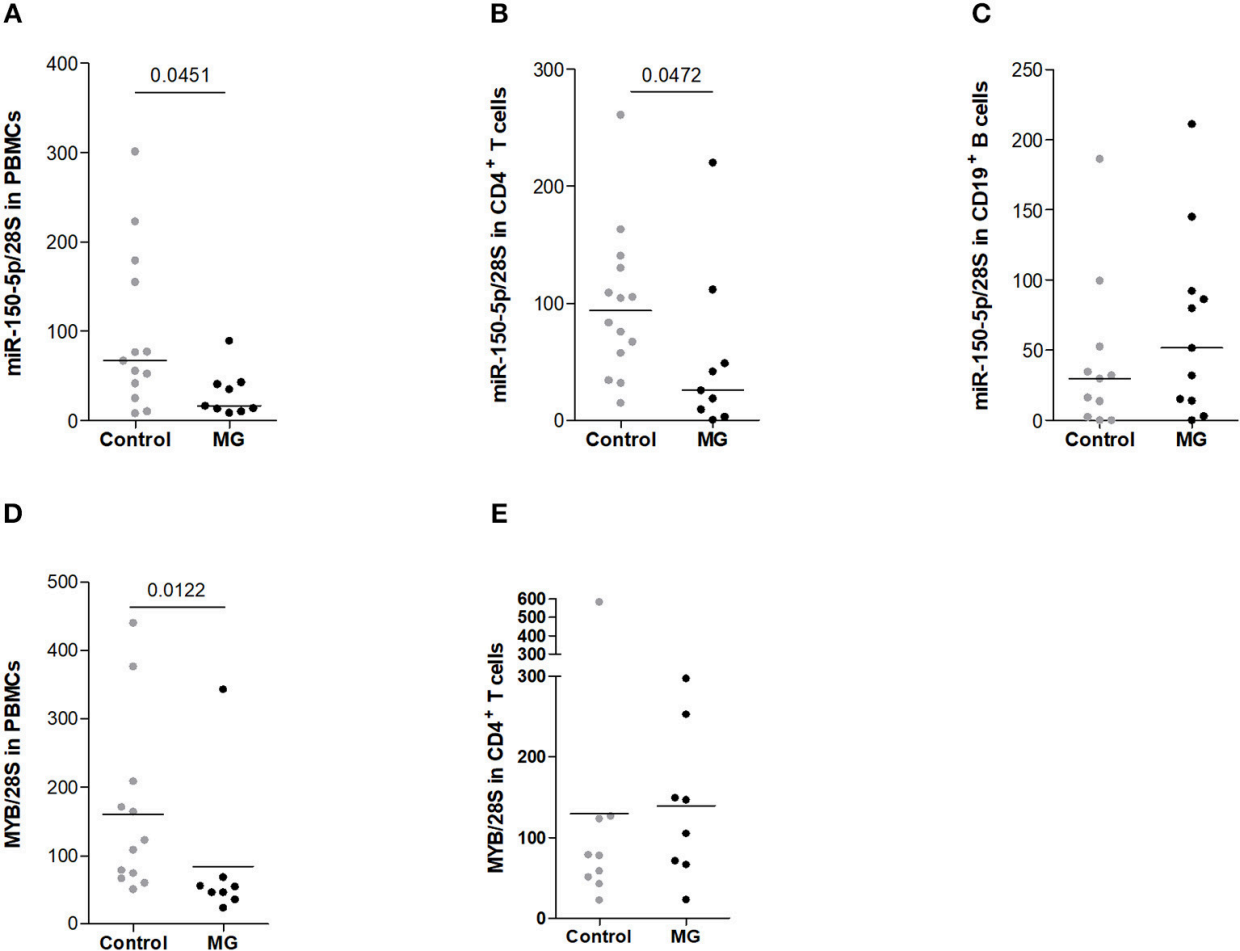
Decreased expression of miR-150-5p in peripheral cells from MG patients. miR-150 expression was assessed by qPCR in **(A)** total PBMCs (controls *n* = 13, MG *n* = 9), **(B)** CD4^+^ T cells (controls *n* = 14, MG *n* = 9) and **(C)** CD19^+^ B cells (controls *n* = 11, MG *n* = 11). miR-150 expression was normalized on 28S expression. **(D)** *MYB* expression was assessed by qPCR in total PBMCs (controls *n* = 12, MG *n* = 8) and **(E)** in CD4^+^ T cells (controls *n* = 9, MG *n* = 8) and normalized on 28S expression. *p*-values were assessed by the Mann-Whitney test.

Circulating miR-150 is overexpressed in the serum of MG patients (10) and we further investigated miR-150 expression in PBMCs from MG patients. Interestingly, miR-150 was found significantly less expressed in MG PBMCs compared to control PBMCs (Figure 3A). Among PBMCs, we investigated the expression of miR-150 in isolated CD4^+^ T cells (Figure 3B) and CD19^+^ B (Figure 3C), the cells mainly involved in the autoimmune response in MG. miR-150 was found decreased in CD4^+^ T cells but not in CD19^+^ B cells (Figures 3B,C). For a few donors, miR-150 expression was also assessed in CD14^+^ monocytes but no difference between MG and control was observed (data not shown). These data suggest that the increase of miR-150 in the serum of MG patients could be linked to a release from CD4^+^ T cells.

Analyzing *MYB* mRNA expression in PBMCs, we were surprised to observe a significant down-regulation of its expression in PBMCs from MG patients but we did not observe the same decrease in CD4^+^ T cells (Figures 3D,E). We did not have enough mRNA samples to properly measure and conclude on MYB in CD19^+^ B cells. To try to better understand the impact of miR-150 on peripheral cells, we then analyzed its direct effects on PBMCs *in vitro*.

### miR-150 Decreased *MYB* Expression in Peripheral Blood Cells

**Figure 4:**
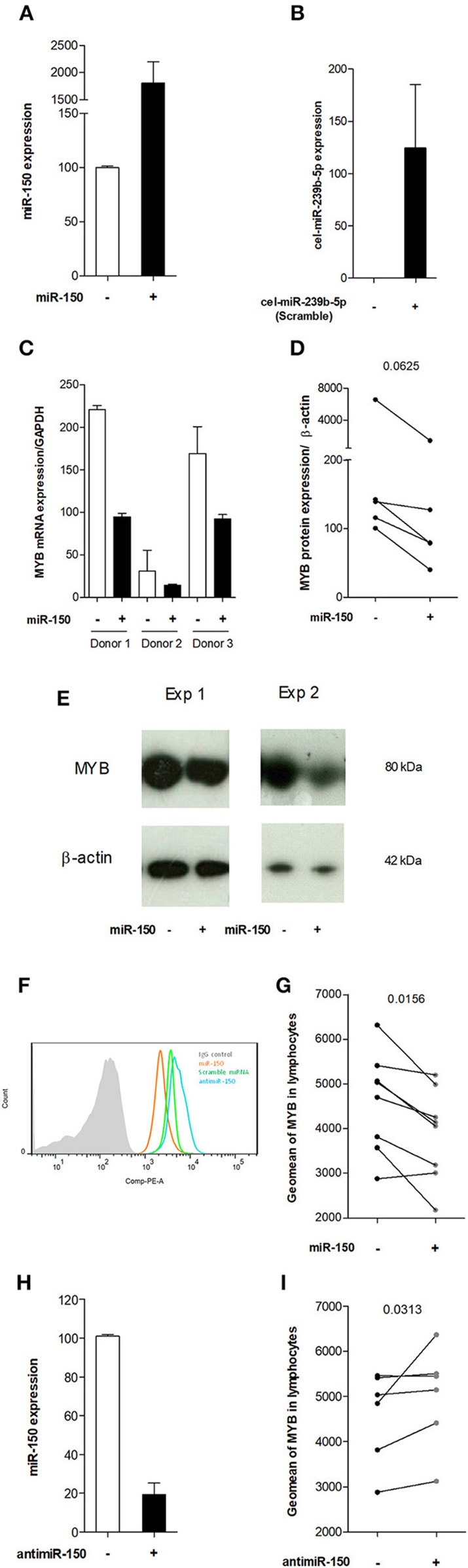
miR-150-5p decreased *MYB* expression in peripheral blood cells *in vitro*. **(A)** Representative experiment of miR-150 expression in PBLs treated with a scramble miRNA (-) or miR-150 (+). **(B)** Representative experiment of cel-miR-293b expression (scramble miRNA) in PBLs treated with miR-150 (-) or a scramble miRNA (+). **(C)** PCR analysis of *MYB* mRNA expression in PBLs treated with a scramble miRNA (-) or miR-150 (+) in 3 different donors. *MYB* mRNA expression was normalized on *GAPDH* mRNA expression. **(D,E)** Western blot analyses of MYB protein expression in PBLs treated with a scramble miRNA (-) or miR-150 (+). **(D)** Western blot quantitative analyses of MYB protein in 5 different donors. MYB expression level was normalized on β-actin. **(E)** Two representative experiments of MYB and β-actin protein expression as seen after Western blot revelation in two different donors. Protein sizes are indicated next to respective bands **(F)** Representative experiment of MYB expression in PBLs treated with miR-150 (orange), scramble miRNA (green) or antimiR-150 (blue), assessed by flow cytometry. Control IgG is represented as a full grey histogram. Cells were first gated for singlet on FSC-H vs. FSC-A, and further analyzed according to their uptake of Live/Dead stain. Finally, lymphocytes were gated on SSC-A *vs*. FSC-A. **(G)** Geomean of MYB expression in the lymphocyte population (*n* = 8), treated with a scramble miRNA (-) or miR-150 (+). **(H)** Representative experiment of miR-150 expression in PBLs treated with a scramble miRNA (-) or antimiR-150 (+). **(I)** Geomean of MYB expression in the lymphocyte population (*n* = 6), treated with a scramble miRNA (-) or antagomiR-150 (+). **(D,G,I)** *p*-values were assessed by the Wilcoxon paired test.

To investigate if circulating miR-150 could have a function in MG and other inflammatory diseases, we analyzed the impact of miR-150 on peripheral circulating cells by treating control PBMCs with miR-150 or with a scramble miRNA from *Caenorhabditis elegans* (cel-miR-293b), as detailed in the method section. To work in physiological conditions, cells were not transfected, and miR-150 and the scramble miRNA were added in cell culture medium and “naturally” delivered into cells thanks to the gymnosis process. Non-adherent PBLs were recovered after 72 h. We validated by qPCR that miR-150 level was elevated intracellularly in cells treated with miR-150 compared to cells treated with the scramble miRNA (Figure 4A). Noteworthy, the efficacy of the gymnosis process was the same for any miRNAs, as the scramble miRNA was also detected within PBMCs after gymnosis (Figure 4B).

Then, we demonstrated at the mRNA (Figure 4C) and protein (Figures 4D,E) levels that MYB was less expressed in cells treated with miR-150. The decreased expression of MYB upon miR-150 treatment was also confirmed by flow cytometry after the exclusion of dying cells and by gating on lymphocytes, as detailed in figure legend (Figures 4F,G). Next, we analyzed by flow cytometry the effect of antimiR-150, at the same concentration than miR-150 and the scramble miRNA, on MYB expression. We confirmed by qPCR that miR-150 expression was decreased in PBLs upon antimiR-150 treatment (Figure 4H). By flow cytometry, we showed that antimiR-150 had an opposite effect compared to miR-150 and significantly increased MYB expression in lymphocytes (Figures 4F–I). Altogether, these results demonstrate that increased level of miR-150 in the serum could penetrate circulating cells and decrease the expression of miR-150 target genes, such as *MYB*. An antimiR-150 treatment by decreasing the levels of endogenous miR-150 could have the opposite effect on target gene expression.

### Increased Cell Death Associated With miR-150 Inhibition

**Figure 5:**
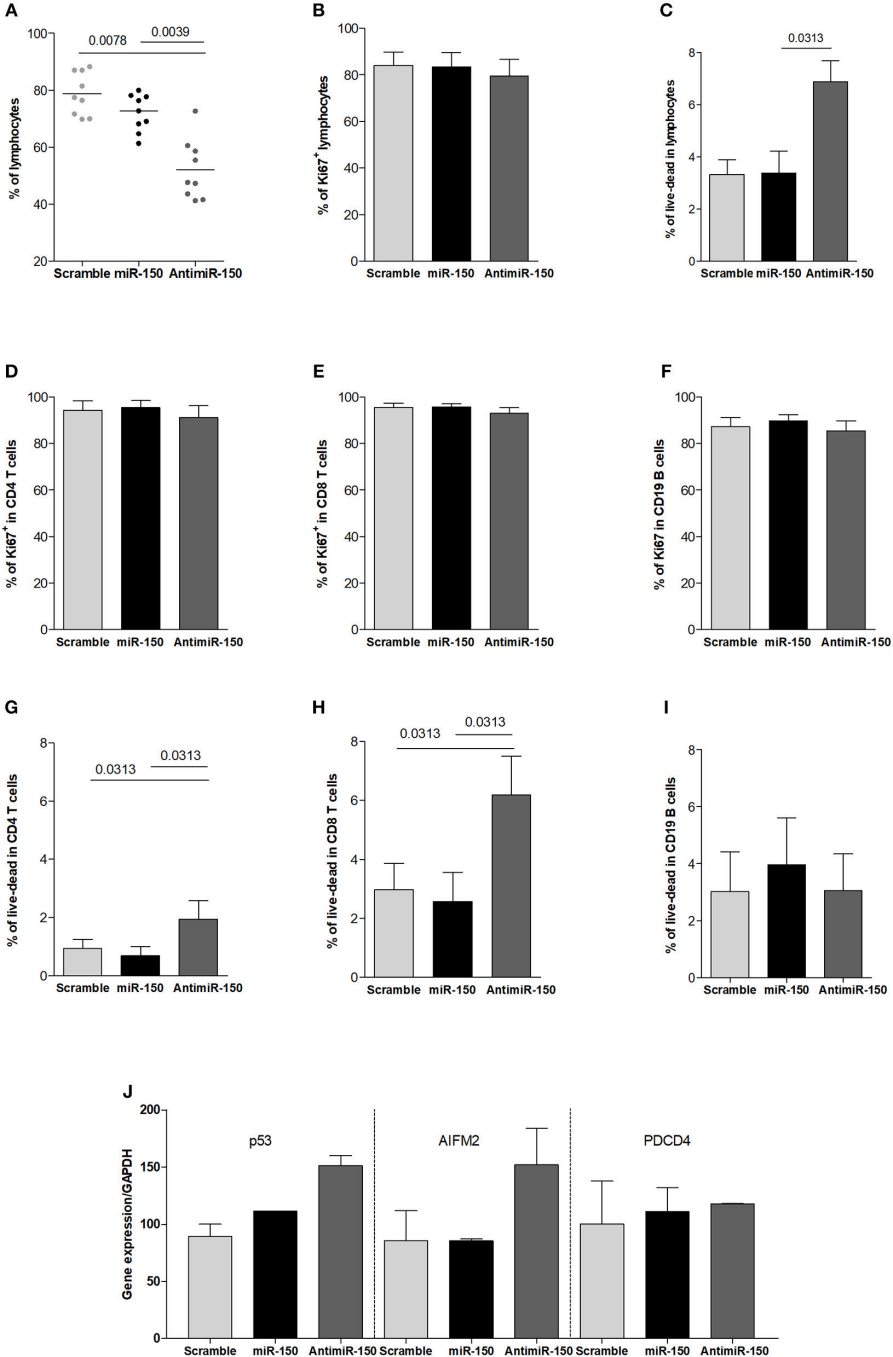
Increased cell death due to miR-150 inhibition. **(A)** Analysis of the effect of miR-150 and antimiR-150 on the percentage of lymphocytes. PBMCs from 9 control donors were cultured for 72 h with a scramble miRNA (10 μM), miR-150 (10 μM) or antimiR-150 (10 μM). PBLs were recovered and analyzed by flow cytometry. Cells were first gated for singlet on FSC-H vs. FSC-A and lymphocytes were gated on SSC-A vs. FSC-A. **(B)** Proliferation was measured in total lymphocytes after 72 h of culture according to Ki67 labeling. **(C)** Cell death was determined in total lymphocytes after 72 h of culture according to a live-dead staining. **(D–F)** To evaluate cell proliferation after 72 h in culture, PBLs were labeled with anti-CD4, CD8, CD19, and Ki67 antibodies (*n* = 5). **(G–I)** To evaluate cell death after 72 h in culture, PBLs were labeled with anti-CD4, CD8, CD19 antibodies and a live-dead stain (*n* = 6). **(J)** qPCR analyses of pro-apoptotic genes P53, AIFM2, and PDCD4 expression in PBLs. Cells from 2 control donors were cultured for 72 h with a scramble miRNA, miR-150 or antimiR-150 (10 μM). Gene expression levels were normalized on GAPDH expression. **(A–I)** *p*-values were assessed by the Wilcoxon paired test.

We also investigated the global effect of miR-150 or antimiR-150 on lymphocytes after 72 h treatments. The addition of miR-150 tends to decrease the proportion of lymphocytes but it was not statistically significant (Figure 5A) and we did not observe any effects of miR-150 on cell proliferation or cell death (Figures 5B,C). In contrast, antimiR-150 significantly decreased the percentage of lymphocytes (Figure 5A). This was associated with an increase of cell death (Figures 5B,C) but not with a decreased proliferation of cells (Figures 5D,F). We next analyzed if CD4^+^ T cells, CD8^+^ T cells and CD19^+^ B cells were similarly impacted by antimiR-150. We observed that antimiR-150 increased with a 2-fold factor CD4^+^ and CD8^+^ T-cell death while it did not affect CD19^+^ B cells (Figures 5G–I).

These results suggested that miR-150 was playing a role in the survival of CD4^+^ and CD8^+^ T cells and the inhibition of miR-150 could induce the expression of pro-apoptotic genes. We identified four pro-apoptotic genes targeted by miR-150 in different prediction databases (miRTarBase, miRWalk, Diana-microT, and TargetScan): *P53* (Tumor Protein 53), *AIFM2* (Apoptosis Inducing Factor Mitochondria associated 2), *SP1* (Specificity Protein 1), and *PDCD4* (Programmed Cell Death 4). We analyzed the effect of antimiR-150 and miR-150 on the expression of these genes. *PDCD4* expression was not modified by any treatments and *SP1* PCR was not effective. However, *P53* and *AIFM2* mRNA expression levels were increased upon the antimiR-150 treatment compared to the scramble miRNA treatment. We did not see any down-regulation of *P53* and *AIFM2* expression upon miR-150 treatment (Figure 5J). This suggests that the basal level of miR-150 in control conditions was sufficient to repress the expression of P53 and AIFM2, and adding more miR-150 did not increase this repression.

As upon miR-150 treatment, we observed a decreased expression of *MYB* (Figure 4) and a slight decrease in the percentage of lymphocytes (Figure 5A), we analyzed the effect of miR-150 on the expression of two anti-apoptotic genes, *BCL-2* (B-cell leukemia/lymphoma 2) and *BCL-XL* (also called BCL2L1: BCL-2 like 2) that are induced by MYB (34). Nevertheless, we did not observe any decreased expression of *BCL-2* and *BCL-XL* mRNAs in PBMCs treated with miR-150 (data not shown). Altogether, these results underline that miR-150 and antimiR-150 did not always have opposite effects and can modulate specifically cell behavior.

## Discussion

We previously showed that miR-150 is increased in the serum of early-onset AChR-MG patients. Its expression is lowered after thymectomy and is correlated with an improvement of the symptoms. Here, we investigated the expression level of miR-150 in the thymus and peripheral blood cells of MG patients to understand why miR-150 is increased in the serum of MG patients and what are the consequences of miR-150 fluctuations on peripheral cells.

### miR-150 Was Overexpressed in Hyperplastic MG Thymuses

Here, we show that miR-150 was more expressed in the thymus of MG patients compared to healthy controls, and in particular in patients displaying a high degree of thymic hyperplasia. Gualeni et al. demonstrated that in reactive lymph nodes, miR-150 is expressed by small lymphocytes of primary follicles and of the mantle zone around GCs (30). In addition, Tan et al. also observed miR-150 to a lesser extent in a few cells in the dark zone of GCs, a dense zone of proliferating B cells (35). We observed a similar expression pattern in ectopic GCs in the thymus of MG patients with miR-150 mainly expressed by B cells of the mantle zone. These observations led us to conclude that the increased expression of miR-150 in hyperplastic MG thymuses was linked to the abnormal presence of B cells and in particular to the development of GCs. We observed that the increased expression of miR-150 in MG thymuses was associated with a decreased expression of *MYB*, the most well-known miR-150 mRNA target that displays four binding sites. MYB is a transcription factor essential for hematopoiesis and is highly expressed in the thymus (36). MYB is also characterized as an early regulator of T-cell associated diseases with an altered expression in autoimmune diseases (37). In the MG thymus, miR-150 could be secreted by B cells and alter *MYB* expression locally and consequently affect T cells, knowing that the T-cell repertoire is altered in MG (38). Indeed, MYB is known to be involved at several stages in thymopoiesis: transition through the double-negative 3 stage, survival of CD4^+^CD8^+^ thymocytes, and differentiation of CD4^+^ thymocytes (39).

As miR-150 is highly expressed in hyperplastic thymuses, the question is whether the serum increase of miR-150 in MG could be linked to a release from the thymus that would explain the serum decrease after thymectomy (10). Indeed, increased expression of miR-150 is often observed both in the serum and inflammatory organs characterized by ectopic lymphoid infiltrations, such as in Sjögren Syndrome (26, 40) and in lupus nephritis (27, 41). To support this hypothesis, we should observe a higher serum level of miR-150 in MG patients with highly hyperplastic thymus. A recent study analyzing the serum levels of miR-150 in MG patients together with the degree of thymic hyperplasia did not observe any correlation (42). However, in this study, 79% of patients were under corticoid therapy. Corticoids strongly induce miR-150 expression in the thymus or in corticoid-sensitive cell lines (19, 20) and even if they are known to decrease the number of thymic GCs, we observed an increased expression of miR-150 in the thymus of cortico-treated MG patients, even with a low or no thymic hyperplasia. We thus analyzed miR-150 levels in serums collected at the time of thymectomy for untreated MG patients with or without thymic hyperplasia. We were not able to analyze miR-150 levels both in the thymus and serum for the same donors. Nevertheless, we did not observe higher serum levels for miR-150 in patients with a highly hyperplastic thymus suggesting that miR-150 serum levels were independent of the degree of thymic hyperplasia.

### miR-150 Is Down-Regulated in CD4^+^ T Cells in MG Patients

In this study, we also showed that miR-150 was less expressed in PBMCs from MG patients compared to controls, demonstrating that circulating and cellular miR-150 levels could vary in opposite ways. This was also observed in other pathological conditions. In HIV/AIDS patients, miR-150 expression is increased in the serum and decreased in PBMCs in symptomatic patients. While the decrease in PBMCs is clearly correlated with the loss of CD4^+^ T cells, the increase in the serum is not well understood (23). In chronic lymphocytic leukemia, Stamatopoulos et al. also observed an increase in serum level of miR-150 and a cellular decrease during disease progression. They suggest that cellular miR-150 could be regulated by its release into the extracellular space (43). In addition, similarly to what we observed in MG, different studies demonstrated that miR-150 is decreased in PBMCs from Sjögren Syndrome patients while it is increased in the serum of patients and in inflammatory salivary glands (26, 40, 44).

Analyzing miR-150 expression in cell subtypes, we observed that the decrease in PBMCs from MG patients was mainly due to CD4^+^ T cells. Similarly, in autoimmune diseases, such as rheumatoid arthritis and systemic lupus erythematosus, miR-150 is less expressed in CD3^+^ T cells, and in particular CD4^+^ T cells (45, 46). In healthy donors, miR-150 was reported to be less expressed in activated CD4^+^ T cells compared to resting cells (47, 48). De Candia et al. demonstrated that upon *in vitro* activation, lymphocytes down-regulate intracellular miR-150 that accumulate in exosomes (18). In addition, they also showed that miR-150 expression level remained practically unchanged in the serum of CD4^+^ T cell-depleted mice after stimulation (28), suggesting that miR-150 expression is mediated through CD4^+^ T cells. Consequently, we hypothesize that increased serum level in MG patients could be due to the release of miR-150 by activated CD4^+^ T cells in MG (49). Indeed, miR-150 belongs to a category of miRNAs that are mainly exported and enriched in extracellular vesicles (50) even if it can also be detected circulating associated with non-vesicular AGO2 ribonucleoprotein complexes (51).

By analyzing MYB expression in PBMCs, we observed that while miR-150 was decreased in PBMCs from MG patients, its main target MYB was also surprisingly down-regulated. Two hypotheses could explain this decrease: (1) PBMCs correspond to a mix of different cell types and changes could correspond to variations in the percentage of a specific cell types. No major changes have never been observed in MG patients regarding the percentage of different cell populations, except a slight decreased proportion of monocytes in MG patients (3). (2) This decrease could reflect the increased serum levels of miR-150 in MG patients (10) that could affect global MYB expression. Of interest, MYB is known to be required for regulatory T cell homeostasis in periphery as *MYB* deletion in these cells resulted in the development of multi-organ autoimmune diseases (52). However, analyzing MYB expression in CD4 T cells we did not observe anymore a decrease in MYB. To better understand the potential impact of miR-150 on peripheral cells, we then analyzed its effects *in vitro* on PBMCs.

### miR-150 Modulated Peripheral Cell Behavior

Most miRNAs described in diseases are characterized only as biomarkers and their functional roles are not further investigated. Here, we analyzed the effects of miR-150 when added on peripheral blood cells. By looking at its major target, MYB, we showed by different approaches that *in vitro* miR-150 was able to decrease *MYB* expression at the mRNA and protein levels. As reviewed by Greig et al., MYB plays a critical and complex role in hematopoietic cells, promoting cell proliferation but also cell differentiation (53). Nevertheless, if the treatment of PBMCs with miR-150 decreased *MYB* expression, in our *in vitro* experiments it did not affect the proliferation or the cellular death of CD4^+^ and CD8^+^ T cells and CD19^+^ B cells.

We also investigated the effect of an antimiR-150, designed to neutralize miR-150 and to cancel its effects. As expected, we demonstrated that antimiR-150 had an antagonistic effect on the expression of *MYB* compared to miR-150. Moreover, we also observed that antimiR-150 induced cellular death of CD4^+^ and CD8^+^ T cells. We showed that two pro-apoptotic genes, *P53* and *AIFM2*, directly regulated by miR-150, were upregulated when cells were treated with antimiR-150. These results suggest that CD4^+^ and CD8^+^ T-cell death could be due to the overexpression of pro-apoptotic genes tightly controlled in the first place by miR-150. This specific effect of antimiR-150 suggest that specific pathways controlled by miR-150 might be involved in T-cell lineage control of apoptosis. Next step would be to investigate if specific T-cell subsets are more sensitive to apoptosis in response to antimiR-150.

Interestingly, *P53* and *AIFM2* were not repressed upon miR-150 treatment. The sensitivity of target genes to the addition of miR-150 or antimiR-150 could be linked to the number of miRNA target sites. Indeed, *MYB* mRNA displays 4 fixation sites for miR-150: its expression is decreased upon miR-150 treatment and increased upon antimiR-150 treatment. However, *P53* and *AIFM2* mRNAs display only one fixation site. Consequently the basal level of miR-150 in control conditions was probably sufficient enough to completely repress the expression of *P53* and *AIFM2*, while the addition of antimiR-150 unlocked the negative control of the expression of these two pro-apoptotic genes.

### Conclusion

miR-150 level is increased in the serum of MG patients and down-regulated after thymectomy, along with an improvement of symptoms (10, 42). In this study, we clearly demonstrated increased expression levels in the thymus of MG patients due to B cells and GC development but the serum levels were not link to the degree of thymic hyperplasia and thymic miR-150 levels. We showed that miR-150 was decreased in peripheral CD4^+^ T cells from MG patients suggesting miR-150 could be released by activated CD4^+^ T cells. After thymectomy, activated T cells decline (49, 54) and that could explained the decreased release of miR-150. Analyzing the impact of miR-150 on peripheral cells, we demonstrated that the basal level of miR-150 had a protective effect on CD4^+^ and CD8^+^ T cells by controlling the expression of pro-apoptotic genes. In addition, increased serum level of miR-150 in MG could play a central role by regulating MYB whose expression is an important regulator of T-cell related autoimmune diseases (37).

## Author Contributions

MC and SM performed and analyzed the experiments, FT collected samples and provided patient information, AVG and AG conducted ISH experiments, EF and JG provided thymic biopsies, AB provided blood samples, SB-A, AVG, AG, and SM read and revised the manuscript, MC and RLP designed the study, analyzed the experiments, and wrote the manuscript.

### Conflict of Interest Statement

The authors declare that the research was conducted in the absence of any commercial or financial relationships that could be construed as a potential conflict of interest.

## Abbreviations

AChR: acetylcholine receptor
AIFM2, apoptosis inducing factor: mitochondria associated 2
GC: germinal center
miRNA: microRNA
miR-150-5p: miR-150
P53: tumor protein 53
PBL: peripheral blood lymphocyte
PBMC: peripheral blood mononuclear cell.

## References

[1] Gilhus NE. Myasthenia gravis. N Engl J Med. (2016) 375:2570–81. 10.1056/NEJMra1602678 28029925

[2] Berrih-Aknin S Ruhlmann N Bismuth J Cizeron-Clairac G Zelman E Shachar I. CCL21 overexpressed on lymphatic vessels drives thymic hyperplasia in myasthenia. Ann Neurol. (2009) 66:521–31. 10.1002/ana.21628 19847900

[3] Weiss JM Cufi P Bismuth J Eymard B Fadel E Berrih-Aknin S. SDF-1/CXCL12 recruits B cells and antigen-presenting cells to the thymus of autoimmune myasthenia gravis patients. Immunobiology. (2013) 218:373–81. 10.1016/j.imbio.2012.05.006 22704519

[4] Corsiero E Nerviani A Bombardieri M Pitzalis C. Ectopic lymphoid structures: powerhouse of autoimmunity. Front Immunol. (2016) 7:430. 10.3389/fimmu.2016.00430 27799933 PMC5066320

[5] Cron MA Maillard S Villegas J Truffault F Sudres M Dragin N. Thymus involvement in early-onset myasthenia gravis. Ann N Y Acad Sci. (2018) 1412:137–45. 10.1111/nyas.13519 29125185

[6] Vincent A Newsom-Davis J. Acetylcholine receptor antibody as a diagnostic test for myasthenia gravis: results in 153 validated cases and 2967 diagnostic assays. J Neurol Neurosurg Psychiatry. (1985) 48:1246–52. 10.1136/jnnp.48.12.1246 4087000 PMC1028609

[7] Truffault F de Montpreville V Eymard B Sharshar T Le Panse R Berrih-Aknin S. Thymic germinal centers and corticosteroids in myasthenia gravis: an immunopathological study in 1035 cases and a critical review. Clin Rev Allergy Immunol. (2017) 52:108–24. 10.1007/s12016-016-8558-3 27273086

[8] Meraouna A Cizeron-Clairac G Panse RL Bismuth J Truffault F Tallaksen C. The chemokine CXCL13 is a key molecule in autoimmune myasthenia gravis. Blood. (2006) 108:432–40. 10.1182/blood-2005-06-2383 16543475 PMC1847364

[9] Wolfe GI Kaminski HJ Aban IB Minisman G Kuo HC Marx A Randomized trial of thymectomy in myasthenia gravis. N Engl J Med. (2016) 375:511–22. 10.1056/NEJMoa1602489 27509100 PMC5189669

[10] Punga T Le Panse R Andersson M Truffault F Berrih-Aknin S Punga AR. Circulating miRNAs in myasthenia gravis: miR-150-5p as a new potential biomarker. Ann Clin Transl Neurol. (2014) 1:49–58. 10.1002/acn3.24 25356381 PMC4207504

[11] Sabre L Maddison P Sadalage G Ambrose PA Punga AR. Circulating microRNA miR-21-5p, miR-150-5p and miR-30e-5p correlate with clinical status in late onset myasthenia gravis. J Neuroimmunol. (2018) 321:164–70. 10.1016/j.jneuroim.2018.05.003 29804819

[12] Zhou B Wang S Mayr C Bartel DP Lodish HF. miR-150, a microRNA expressed in mature B and T cells, blocks early B cell development when expressed prematurely. Proc Natl Acad Sci USA. (2007) 104:7080–5. 10.1073/pnas.0702409104 17438277 PMC1855395

[13] Xiao C Calado DP Galler G Thai T-H Patterson HC Wang J. MiR-150 Controls B cell differentiation by targeting the transcription factor c-Myb. Cell. (2007) 131:146–59. 10.1016/j.cell.2007.07.021 17923094

[14] Smith NL Wissink EM Grimson A Rudd BD. miR-150 regulates differentiation and cytolytic effector function in CD8+ T cells. Sci Rep. (2015) 5:16399. 10.1038/srep16399 26549197 PMC4637875

[15] Kroesen B-J Teteloshvili N Smigielska-Czepiel K Brouwer E Boots AMH van den Berg A. Immuno-miRs: critical regulators of T-cell development, function and ageing. Immunology. (2014) 144:1–10. 10.1111/imm.12367 25093579 PMC4264905

[16] Seo KH Zhou L Meng D Xu J Dong Z Mi QS. Loss of microRNAs in thymus perturbs invariant NKT cell development and function. Cell Mol Immunol. (2010) 7:447–53. 10.1038/cmi.2010.49 20852654 PMC4002964

[17] Zhou L Park JJ Zheng Q Dong Z Mi Q. MicroRNAs are key regulators controlling iNKT and regulatory T-cell development and function. Cell Mol Immunol. (2011) 8:380–7. 10.1038/cmi.2011.27 21822298 PMC4012887

[18] de Candia P Torri A Gorletta T Fedeli M Bulgheroni E Cheroni C. Intracellular modulation, extracellular disposal and serum increase of MiR-150 mark lymphocyte activation. PLoS ONE. (2013) 8:e75348. 10.1371/journal.pone.0075348 24205408 PMC3805464

[19] Belkaya S Silge RL Hoover AR Medeiros JJ Eitson JL Becker AM. Dynamic modulation of thymic MicroRNAs in response to stress. PLoS ONE. (2011) 6:e27580. 10.1371/journal.pone.0027580 22110677 PMC3217971

[20] Palagani A Op de Beeck K Naulaerts S Diddens J Sekhar Chirumamilla C Van Camp G. Ectopic MicroRNA-150-5p transcription sensitizes glucocorticoid therapy response in MM1S multiple myeloma cells but fails to overcome hormone therapy resistance in MM1R Cells. PLoS ONE. (2014) 9:e113842. 10.1371/journal.pone.0113842 25474406 PMC4256227

[21] Gandhi R Healy B Gholipour T Egorova S Musallam A Hussain MS. Circulating microRNAs as biomarkers for disease staging in multiple sclerosis. Ann Neurol. (2013) 73:729–40. 10.1002/ana.23880 23494648

[22] Fourie NH Peace RM Abey SK Sherwin LB Rahim-Williams B Smyser PA. Elevated circulating miR-150 and miR-342-3p in patients with irritable bowel syndrome. Exp Mol Pathol. (2014) 96:422–5. 10.1016/j.yexmp.2014.04.009 24768587 PMC4119883

[23] Munshi SU Panda H Holla P Rewari BB Jameel S. MicroRNA-150 Is a potential biomarker of HIV/AIDS disease progression and therapy. PLoS ONE. (2014) 9:e95920. 10.1371/journal.pone.0095920 24828336 PMC4020752

[24] Wang S Yin J Li T Yuan L Wang D He J. Upregulated circulating miR-150 is associated with the risk of intrahepatic cholangiocarcinoma. Oncol Rep. (2015) 33:819–25. 10.3892/or.2014.3641 25482320

[25] Ma Y Zhang P Wang F Zhang H Yang J Peng J. miR-150 as a potential biomarker associated with prognosis and therapeutic outcome in colorectal cancer. Gut. (2012) 61:1447–53. 10.1136/gutjnl-2011-301122 22052060

[26] Alevizos I Alexander S Turner RJ Illei GG. MicroRNA expression profiles as biomarkers of minor salivary gland inflammation and dysfunction in Sjogren's syndrome. Arthritis Rheum. (2011) 63:535–44. 10.1002/art.30131 21280008 PMC3653295

[27] Zhou H Hasni SA Perez P Tandon M Jang SI Zheng C. miR-150 promotes renal fibrosis in lupus nephritis by downregulating SOCS1. J Am Soc Nephrol. (2013) 24:1073–87. 10.1681/ASN.2012080849 23723424 PMC3699828

[28] de Candia P Torri A Pagani M Abrignani S. Serum microRNAs as biomarkers of human lymphocyte activation in health and disease. Front Immunol. (2014) 5:43. 10.3389/fimmu.2014.00043 24575093 PMC3918657

[29] Dragin N Bismuth J Cizeron-Clairac G Biferi MG Berthault C Serraf A. Estrogen-mediated downregulation of AIRE influences sexual dimorphism in autoimmune diseases. J Clin Invest. (2016) 126:1525–37. 10.1172/JCI81894 26999605 PMC4811157

[30] Gualeni AV Volpi CC Carbone A Gloghini A. A novel semi-automated *in situ* hybridisation protocol for microRNA detection in paraffin embedded tissue sections. J Clin Pathol. (2015) 68:661–4. 10.1136/jclinpath-2015-203005 25934842

[31] English D Andersen BR. Single-step separation of red blood cells. granulocytes and mononuclear leukocytes on discontinuous density gradients of ficoll-hypaque. J Immunol Methods. (1974) 5:249–52. 10.1016/0022-1759(74)90109-4 4427075

[32] Takahashi M Contu VR Kabuta C Hase K Fujiwara Y Wada K. SIDT2 mediates gymnosis, the uptake of naked single-stranded oligonucleotides into living cells. RNA Biol. (2017) 14:1534–43. 10.1080/15476286.2017.1302641 28277980 PMC5785214

[33] Cron MA Maillard S Delisle F Samson N Truffault F Foti M. Analysis of microRNA expression in the thymus of myasthenia gravis patients opens new research avenues. Autoimmun Rev. (2018) 17:588–600. 10.1016/j.autrev.2018.01.008 29655674

[34] Chen Z Stelekati E Kurachi M Yu S Cai Z Manne S. miR-150 regulates memory CD8 T cell differentiation via c-Myb. Cell Rep. (2017) 20:2584–97. 10.1016/j.celrep.2017.08.060 28903040 PMC5611819

[35] Tan LP Wang M Robertus JL Schakel RN Gibcus JH Diepstra A. miRNA profiling of B-cell subsets: specific miRNA profile for germinal center B cells with variation between centroblasts and centrocytes. Lab Invest. (2009) 89:708–16. 10.1038/labinvest.2009.26 19349957

[36] Wang X Angelis N Thein SL. MYB - a regulatory factor in hematopoiesis. Gene. (2018) 665:6–17. 10.1016/j.gene.2018.04.065 29704633 PMC10764194

[37] Gustafsson M Gawel DR Alfredsson L Baranzini S Bjorkander J Blomgran R. A validated gene regulatory network and GWAS identifies early regulators of T cell-associated diseases. Sci Transl Med. (2015) 7:313ra178. 10.1126/scitranslmed.aad2722 26560356

[38] Truffault F Cohen-Kaminsky S Khalil I Levasseur P Berrih-Aknin S. Altered intrathymic T-cell repertoire in human myasthenia gravis. Ann Neurol. (1997) 41:731–41. 10.1002/ana.410410609 9189034

[39] Bender TP Kremer CS Kraus M Buch T Rajewsky K. Critical functions for c-Myb at three checkpoints during thymocyte development. Nat Immunol. (2004) 5:721–9. 10.1038/ni1085 15195090

[40] Lopes AP Hillen MR Chouri E Blokland SLM Bekker CPJ Kruize AA. Circulating small non-coding RNAs reflect IFN status and B cell hyperactivity in patients with primary Sjogren's syndrome. PLoS ONE. (2018) 13:e0193157. 10.1371/journal.pone.0193157 29447268 PMC5814054

[41] Li H Ding G. Elevated serum inflammatory cytokines in lupus nephritis patients, in association with promoted hsa-miR-125a. Clin Lab. (2016) 62:631–8. 10.7754/Clin.Lab.2015.150812 27215082

[42] Molin CJ Sabre L Weis C-A Punga T Punga AR. Thymectomy lowers the myasthenia gravis biomarker miR-150-5p. Neurol Neuroimmunol Neuroinflamm. (2018) 5:e450. 10.1212/NXI.0000000000000450 29511707 PMC5833334

[43] Stamatopoulos B Van Damme M Crompot E Dessars B El Housni H Mineur P. Opposite prognostic significance of cellular and serum circulating microRNA-150 in chronic lymphocytic leukemia patients. Mol Med. (2015) 21:123–33. 10.2119/molmed.2014.00214 25584781 PMC4461585

[44] Chen JQ Papp G Poliska S Szabo K Tarr T Balint BL. MicroRNA expression profiles identify disease-specific alterations in systemic lupus erythematosus and primary Sjogren's syndrome. PLoS ONE. (2017) 12:e0174585. 10.1371/journal.pone.0174585 28339495 PMC5365120

[45] Li J Wan Y Guo Q Zou L Zhang J Fang Y. Altered microRNA expression profile with miR-146a upregulation in CD4+ T cells from patients with rheumatoid arthritis. Arthritis Res Ther. (2010) 12:R81. 10.1186/ar3006 20459811 PMC2911863

[46] Lu MC Lai NS Chen HC Yu HC Huang KY Tung CH. Decreased microRNA(miR)-145 and increased miR-224 expression in T cells from patients with systemic lupus erythematosus involved in lupus immunopathogenesis. Clin Exp Immunol. (2013) 171:91–9. 10.1111/j.1365-2249.2012.04676.x 23199328 PMC3530100

[47] Huang J Wang F Argyris E Chen K Liang Z Tian H. Cellular microRNAs contribute to HIV-1 latency in resting primary CD4+ T lymphocytes. Nat Med. (2007) 13:1241–7. 10.1038/nm1639 17906637

[48] King BC Esguerra JL Golec E Eliasson L Kemper C Blom AM. CD46 activation regulates miR-150-mediated control of GLUT1 expression and cytokine secretion in human CD4+ T cells. J Immunol. (2016) 196:1636–45. 10.4049/jimmunol.1500516 26746193 PMC4745132

[49] Ahlberg R Yi Q Pirskanen R Matell G Sundevall AC Aberg B. The effect of thymectomy on autoreactive T- and B-lymphocytes in myasthenia gravis. J Neuroimmunol. (1997) 74:45–54. 10.1016/S0165-5728(96)00204-4 9119978

[50] Arroyo JD Chevillet JR Kroh EM Ruf IK Pritchard CC Gibson DF. Argonaute2 complexes carry a population of circulating microRNAs independent of vesicles in human plasma. Proc Natl Acad Sci USA. (2011) 108:5003–8. 10.1073/pnas.1019055108 21383194 PMC3064324

[51] Guduric-Fuchs J O'Connor A Camp B O'Neill CL Medina RJ Simpson DA. Selective extracellular vesicle-mediated export of an overlapping set of microRNAs from multiple cell types. BMC Genomics. (2012) 13:357. 10.1186/1471-2164-13-357 22849433 PMC3532190

[52] Dias S D'Amico A Cretney E Liao Y Tellier J Bruggeman C. Effector regulatory T cell differentiation and immune homeostasis depend on the transcription factor Myb. Immunity. (2017) 46:78–91. 10.1016/j.immuni.2016.12.017 28099866

[53] Greig KT Carotta S Nutt SL. Critical roles for c-Myb in hematopoietic progenitor cells. Semin Immunol. (2008) 20:247–56. 10.1016/j.smim.2008.05.003 18585056

[54] Huang YM Pirskanen R Giscombe R Link H Lefvert AK. Circulating CD4+CD25+ and CD4+CD25+ T cells in myasthenia gravis and in relation to thymectomy. Scand J Immunol. (2004) 59:408–14. 10.1111/j.0300-9475.2004.01410.x 15049785

